# Unconscious Processing Contaminates Objective Measures of Conscious Perception: Evidence From the Liminal Prime Paradigm

**DOI:** 10.5334/joc.402

**Published:** 2024-10-01

**Authors:** Nitzan Micher, Diana Mazenko, Dominique Lamy

**Affiliations:** 1 School of Psychological Sciences, Tel Aviv University, Tel Aviv, Israel; 2 Sagol School of Neuroscience, Tel Aviv University, Tel Aviv, Israel

**Keywords:** Consciousness, Visual perception, Visual word processing

## Abstract

Assessing unconscious processing requires a valid measure of conscious perception. However, the two measures most commonly used, subjective reports and forced-choice discrimination, do not always converge: observers can discriminate stimuli rated as invisible better than chance. A debated issue is whether this phenomenon indicates that subjective reports of unawareness are contaminated by conscious perception, or that forced-choice discrimination performance is contaminated by unconscious processing. To address this question, we took advantage of a previously reported dissociation using masked response priming: for primes rated as invisible on a multi-point scale, response priming occurs only for fast trials, whereas for consciously perceived primes, response priming occurs across response times. Here, we replicated this dissociation, confirming that invisibility-reports were not contaminated by conscious perception. Crucially, we measured prime-discrimination performance within the same experiment and found above-chance performance for unseen primes. Together, these findings suggest that forced-choice discrimination performance is contaminated by unconscious processing.

## 1. Introduction

To what extent can our brains extract information from stimuli that we are not aware of? The notion that some mental processes operate beneath the surface of conscious awareness has long drawn psychologists’ interest (e.g., Freud, 1899; James, 1890). Yet, extensive empirical investigation of unconscious processes took off only from the middle of the 20^th^ century (Baker, 1938; Eriksen, 1960; Greenwald et al., 1996; Hilgard et al., 1941; Holender, 1986; Marcel, 1983; Merikle, 1982; Taylor, 1953). Despite decades of research, the field still attempts to overcome the same methodological issues it has tackled in the previous century (Gelbard-Sagiv et al., 2016; Kiefer et al., 2023; Lamy et al., 2019; Lin & Murray, 2014; Meyen et al., 2021; Michel, 2022; Peters & Lau, 2015; Sandberg et al., 2022; Shanks, 2017; Vadillo et al., 2021; Wierzchoń, Szczepanowski, et al., 2014). Perhaps the most debated issue concerns which measures are valid for demonstrating unconscious processing. Specifically, should (absence of) awareness be measured using a subjective or an objective measure? The goal of the present study was to address this question.

Subjective measures of conscious perception rely on observers’ introspection about the visibility or clarity of presented stimuli: participants are held to be subjectively unaware of a stimulus if they report having no experience of that stimulus (Eriksen, 1960; Merikle, 1984; Sandberg et al., 2011; Timmermans & Cleeremans, 2014). Objective measures of conscious perception, on the other hand, rely on participants’ ability to discriminate some property of a stimulus, for instance, whether a shape is a square or a circle. Participants are held to be objectively unaware of the stimulus’ property when their discrimination performance is at chance (Dehaene et al., 1998; Holender, 1986; Marcel, 1983; Merikle, 1982; Merikle et al., 2001).

Some studies indicated that the two measures of awareness diverge, that is, that participants can discriminate a stimulus property above chance while reporting having no subjective experience of the stimulus (Adams, 1957; Eriksen, 1960; Meeres & Graves, 1990; Merikle et al., 2001; Miller, 1939; Sidis, 1898). This “blindsight” phenomenon is often observed in neurological patients (Poppel et al., 1973; Sanders et al., 1974; Smits et al., 2019; Stoerig et al., 1993; Weiskrantz, 1990, 1996, 2004) but also in healthy observers (Boyer et al., 2005; Derda et al., 2019; Fu et al., 2017; Koivisto & Grassini, 2016; Lamy et al., 2009; Ro et al., 2004; Salti et al., 2012; Sandberg et al., 2010, 2011; Soto et al., 2011).

Why does blindsight-like performance^1^ occur? This question is at the heart of the subjective-objective measures dispute because it shows a divorce between the two measures. If we take observers’ subjective reports seriously, then people who exhibit blindsight-like performance are truly unaware of the stimuli and they discriminate them above chance because they process them unconsciously (Hesselmann et al., 2011; Merikle et al., 2001; Michel, 2022; Sidis, 1898). Conversely, if we adopt the view that only chance discrimination performance attests that a stimulus is not consciously perceived, then blindsight-like performance indicates that participants are in fact aware of the stimuli; they report being unaware of them because they adopt a conservative response criterion (this is known as the criterion problem, see e.g., Eriksen, 1960). In other words, participants miscategorize consciously perceived stimuli as “unseen” and it is conscious processing of these stimuli that produces above-chance discrimination. This is not to say that proponents of objective measures deny the subjective character of conscious experience. They only claim that reports of this experience are susceptible to biases, that is, false reports of invisibility, and that relying on objective measures addresses this problem.

Note that even if unconscious processing contaminates forced-choice discrimination performance, ensuring that such performance it at chance should guarantee that the stimulus is both subjectively and objectively invisible. This is the rationale that underlies the subliminal-prime paradigm. However, several criticisms undermine the validity of the objective measure of awareness in that paradigm.

In a typical “subliminal-prime” experiment, participants first categorize targets that appear shortly after primes, which can be either congruent with the target (i.e., associated with the same response as the target) or incongruent (i.e., associated with the alternative response). Better target categorization on congruent than on incongruent trials (or response priming) attests that the prime was processed and influenced responses to the target. Then, participants take a prime-awareness test that is similar to the priming phase, except that instead of responding to the target, they have to discriminate some property of the prime. Chance performance on the awareness test is taken to ensure that participants were also unaware of the primes in the priming phase and that any response priming during that phase reflected unconscious processing.

Several authors have suggested that the subliminal-prime paradigm may underestimate the contribution of conscious processes. First, the fact that response priming and prime awareness are measured under different task conditions raises the task-comparability problem (e.g., Lin & Murray, 2014; Pratte & Rouder, 2009; Reingold & Merikle, 1988, 1990). For instance, Pratte and Rouder (2009) noted that as the task is more difficult in the priming than in the awareness-test phase, participants are less attentive in the former. Accordingly, these authors reported that for the same stimuli, prime discrimination performance is at chance when all stimuli are degraded but becomes better than chance when participants are motivated to remain on task by introducing high-visibility primes among the degraded primes. Second, the target is likely to contaminate responses to the prime in the awareness test phase, because (a) it is much more salient than the prime, (b) it is associated with the same potential responses, (c) participants have repeatedly attended and responded to it in the preceding priming phase. Such contamination should push prime-discrimination performance towards chance since the targets are randomly congruent or incongruent with the primes (see Peremen & Lamy, 2014; Vermeiren & Cleeremans, 2012). This criticism also applies when priming and prime awareness are measured on each trial to address the task comparability problem (e.g., Koivisto & Rientamo, 2016). Again, responses to the highly visible targets are likely to contaminate the responses to the weakly perceived primes.

One way to address both caveats is to measure priming and conscious perception in the same phase but on different trials (e.g., Faivre et al., 2012). Participants respond to the target on most trials, and on the remaining trials the target is replaced with a symbol that indicates that participants should discriminate the prime instead. Crucially, participants do not know which stimulus they have to categorize (target or prime) until the target display is presented. Thus, prime discrimination performance and priming can be measured under the same conditions. Interestingly, this approach has not yielded evidence for unconscious processing (see Faivre et al., 2012).

To summarize, while many studies that relied on the subliminal-prime paradigm reported significant response-priming effects (Bruno et al., 2020; Kiesel et al., 2009; Koivisto & Rientamo, 2016; Ortells et al., 2016), these effects might reflect conscious rather than unconscious processing because conscious perception was underestimated. As a result, unconscious processing may be far more limited in scope than previously thought (e.g., Faivre, 2012). However, the requirement that discrimination be at chance to ensure invisibility also underestimates unconscious processing. Indeed, to prevent above-chance discrimination, researchers use highly degraded stimuli, far more degraded than needed to obtain subjective “unseen” reports. If unconscious processing requires stronger signals that produce above-chance discrimination, then the chance-discrimination standard may be too strict, and unconscious processing may be missed. To illustrate this point *ad absurdum*, blindfolding observers would be highly effective in preventing participants from discriminating stimuli better than chance, but it would also obviously eliminate any unconscious processing (Peremen & Lamy, 2014). As is clear from the foregoing discussion, then, arbitrating between the two interpretations of blindsight-like performance is essential to advance our understanding of unconscious processing.

Here, we propose to test the claim that blindsight-like performance can reflect unconscious processing by taking advantage of a dissociation between subjectively conscious and unconscious processing observed in our lab using the *liminal-prime paradigm* (Avneon & Lamy, 2018, 2019; Kimchi et al., 2018; Lamy et al., 2015, 2019; Micher & Lamy, 2023; Ophir et al., 2018; 2020; Peremen et al., 2013; Sabary et al., 2020): we found conscious and unconscious processing to be dissociated by their time courses on the reaction-times distribution.

The main motivation of the liminal-prime paradigm is to assess the extent to which a given process depends on conscious perception by comparing the impact of unseen versus seen stimuli on behavior. For instance, in Micher and Lamy’s (2023) study, participants were first asked to discriminate whether a target was the name of a small or a large animal (relative to a shoebox). Unlike in the subliminal-prime paradigm, however, the prime was liminal,^2^ that is, its duration elicited a variable subjective experience, from no experience at all to a clear experience. Participants were then asked to rate the visibility of the prime using a 4-point visibility rating scale, similar to the Perceptual Awareness Scale (henceforth, “PAS”, Ramsøy & Overgaard, 2004). Primes were labeled as “unseen” when participants selected the lowest rating and as “seen” when they selected the highest rating. The outcomes of interest were (1) whether compatibility between the responses associated with the unseen primes and the visible targets affected performance and (2) how this unaware response priming effect compared to aware response priming. The findings showed significant unaware response priming that was smaller than aware response priming.

Since the liminal-prime paradigm relies on a subjective measure of awareness, the criterion argument against the claim that the prime was truly unseen may still hold. Accordingly, both aware and unaware response priming may reflect different degrees of conscious processing. However, a dissociation between aware and unaware response priming across the RT distribution renders this possibility unlikely (Avneon & Lamy, 2018, 2019; Micher & Lamy, 2023). Specifically, we found that while aware response priming was robust on both fast and slow trials, unaware response priming occurred only on the fastest trials and disappeared on trials with slower responses (see Dehaene et al., 1998, Figure 2 for a similar finding). Had unaware response priming emanated from a few conscious-prime trials (erroneously labeled as unseen), we should have observed unaware response priming also on slow-response trials, but we did not. We therefore conclude that contamination of “unseen” reports by conscious perception was minimal, if at all present.

In the present study, we relied on this dissociation to test whether above-chance discrimination of subjectively unseen stimuli reflects unconscious processing or conscious processing. The experiment was similar to Micher and Lamy’s (2023) first experiment where the primes and targets were drawn from the same set of four animal names. On most trials, participants categorized the target and then rated the visibility of the liminal prime (target-categorization trials). On the remaining trials, the target was replaced with a symbol prompting participants to categorize the prime instead of the target (prime-categorization trials, see Faivre et al., 2012) and again, participants rated prime visibility. This design allowed us to measure both response priming and prime-categorization performance as a function of prime visibility, in the same experiment.

Because participants did not know whether they would have to categorize the target or the prime until the target display appeared, they should process the prime in the same way in both trial types. We reasoned that observing the dissociation between unaware and aware response priming on target-categorization trials would indicate that conscious perception did not contaminate the subjective “unseen” reports on both target- and prime-categorization trials. Therefore, finding that discrimination of primes rated as “unseen” (i.e., blindsight-like performance) is above-chance would reflect unconscious processing rather than conscious processing. We would conclude that objective measures of conscious perception are contaminated by unconscious processing.

We examined the dissociation between conscious and unconscious processing by measuring response priming on the 50% fastest and the 50% slowest trials. Unlike in our previous study (Micher & Lamy, 2023), we did so for each visibility rating. Our rationale was that if the dissociation indeed occurs between unconscious and conscious percepts – rather than between degraded and clear percepts – the impact of stimuli that are not fully perceived should also be observed on slow trials. Accordingly, we expected response priming to occur only on fast trials for 0-visibility rated primes and on both fast and slow trials for 3-visibility rated primes as well as for 2-visibility rated trials. For 1-visibility trials, we had no clear prediction, because during debriefing, participants often report using this rating when they are off-task or blink (see Avneon & Lamy, 2019; Kimchi et al., 2018; Ophir et al., 2018 for related observations). We also expected accuracy at categorizing the prime to increase as reported visibility increased. Crucially, we expected performance to be above chance on 0-visibility trials (see Sandberg et al., 2010, for above-chance discrimination of masked targets at liminal stimulus-exposure levels).

Taken together, such a pattern of results would support the hypothesis that blindsight-like performance reflects unconscious processing rather than contamination of subjective reports by conscious perception. By contrast, finding chance prime-discrimination performance on 0-visibility trials would not allow us to answer our research question. However, such a finding would suggest that blindsight-like performance may occur only for very simple discriminations (e.g., simple shape discrimination, Sandberg et al., 2010) or that previous reports of such performance might have been contaminated by conscious perception. Thus, it would call for further research to establish blindsight-like performance for higher-level discriminations.

## 2. Methods

The study’s method, planned sample, exclusion criteria, hypotheses, and statistical analyses were pre-registered on OSF (https://osf.io/my5kb).^3^

### 2.1. Sample-size selection

We selected a sample size of 40 participants based on Micher and Lamy (2023, Exp. 1, “used” primes). Their data suggest that a sample size of 40 is sufficient to find decisive evidence for conscious and unconscious response priming on fast-response trials (BF_10_ > 100) and substantial evidence against unconscious response priming on slow-response trials (BF_01_ = 7.79).

### 2.2. Participants

Forty students (24 females, mean age = 24.23, SD = 2.17) participated in the experiment for course credit or payment (40 New Israeli Shekels). All reported normal or corrected-to-normal vision, were native Hebrew speakers, and had no ADHD condition.

### 2.3. Apparatus

The experiment took place in a dimly lit room. All stimuli were presented on a screen using 1920 × 1280 resolution graphics mode and 120-Hz refresh rate. The experimental procedure was programmed in PsychoPy (Peirce et al., 2019). Responses were collected via the computer keyboard. The viewing distance was set at approximately 50 cm from the monitor.

### 2.4. Stimuli

The fixation was a 0.5° × 0.5° plus sign (+). The target and prime stimuli were selected from the same set of eight animal names (four small animals and four large animals) used by Micher and Lamy (2023, Exp. 1). They were all 4-letter Hebrew words. For each participant, four animal words (two large and two small) could serve both as targets and as primes. The small-animal names were “נמלה” (ant), “יתוש” (mosquito), “זבוב” (fly), and “פרפר” (butterfly), and the large-animal names were “אריה” (lion), “כבשה” (sheep), “שועל” (fox), and “חמור” (donkey). On prime-categorization trials, the letter string ‘WWWWW’ appeared instead of a target word. The forward and backward masks consisted of strings of 5 symbols (‘#@#@# and ‘@#@#@’, respectively). All stimuli were drawn in a 28-point Arial font (~1.15° of visual angle) and were enclosed in a frame (14.2 × 2.8° of visual angle, 1-pixel thick). All stimuli were gray (RGB: 127, 127, 127, with a luminance of about 33 cd/m^2^) and were centered at fixation.

### 2.5. Procedure

**Figure 1:**
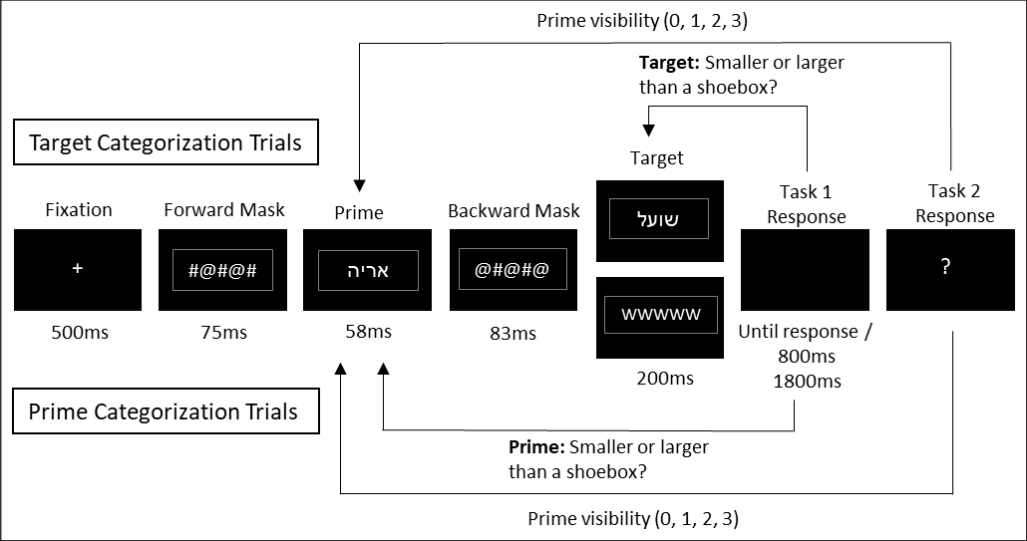
Sequence of events on each trial. On target-categorization trials participants first determined whether the target word represented an animal smaller or larger than a shoebox. Then, they rated the prime’s visibility on a 4-level scale. Prime-categorization trials were similar, except that participants categorized the *prime* instead of the *target* animal name. Stimuli are not drawn to scale.

The sequence of events is shown in Figure 1. The experiment consisted of two types of trials: target-categorization trials and prime-categorization trials. Every trial consisted of the successive presentation of a fixation (500 ms), forward mask (75 ms), prime (58 ms), and backward mask (83 ms). Then, either a target (on target-categorization trials) or the “WWWWW” string (on prime-categorization trials) appeared for 200 ms, followed by a blank that appeared for 800 ms on target-categorization trials and for 1,800 ms on prime-categorization trials, or until the first response was given. A question mark immediately followed and remained on the screen until the second response was given.

**Figure 2:**
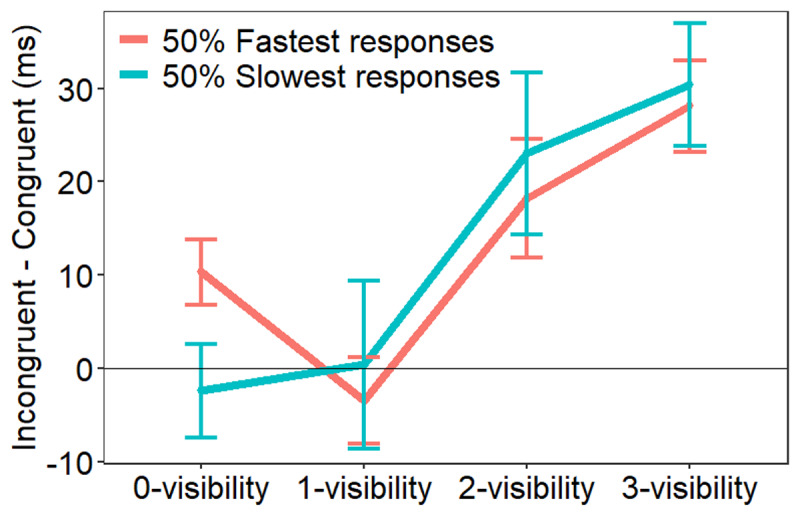
Mean response priming on RTs (in milliseconds) as a function of prime’s visibility rating and response speed (50% fastest vs. 50% slowest). Error bars indicate standard errors.

On each trial, participants provided two responses: a categorization-task response and a visibility report. On target-categorization trials, participants categorized the *target* word as an animal larger or smaller than a shoebox by pressing designated keys (right or left arrow-key, respectively) with their right hands as fast and as accurately as possible. When they incorrectly categorized the target or failed to give a response in the allotted time, they heard a 150 ms 1000-Hz error beep. On prime-categorization trials, the ‘WWWWW’ string prompted participants to perform the animal-categorization task on the *prime* and to guess if they could not tell; unlike on target-discrimination trials, participants received no feedback for errors. On both target- and prime-categorization trials, the second response was a non-speeded rating of the subjective visibility of the prime: participants were asked to use a scale ranging from 0 to 3, by pressing one of four different designated keys (z, x, c, and v for 0, 1, 2 and 3, respectively) with their left hands. Specifically, they were instructed to use “0” if they did not see anything on the screen before target onset (other than the masks), “1” if they saw a glimpse or no more than one individual letter, “2” if they saw several letters but could not say what word they might have formed and “3” if they saw a word and could name it.^4^

### 2.6. Design

The experiment began with five practice blocks of 20 trials each, aimed at familiarizing participants with the tasks. These were followed by 960 experimental trials divided into 8 blocks separated by self-paced breaks. In the first practice block, participants were only asked to perform the target-categorization task. At the end of the first block, participants were informed about the presence of the primes and were given instructions about how to use the visibility ratings. In the second and third practice blocks, trials were similar to target-categorization trials and prime-categorization trials, respectively, except that the prime appeared for 200 ms. As primes were clearly visible, we could verify that participants understood our instructions and most often used 3-visibility ratings on prime-present trials, and 0-visbility ratings on prime-absent trials. The fourth and fifth practice blocks were randomly drawn from the experimental trials.

The experiment included target-categorization trials where the prime was present (60% of all experimental trials), target-categorization trials where the prime was absent (henceforth, catch trials, 20%) and prime-categorization trials (20%). On each trial, the prime and target identities were randomly selected and were equally likely to be congruent (i.e., the prime and target were both associated with the same response, e.g., ‘ant’ and ‘fly’) or incongruent (i.e., the prime and target were associated with different responses, e.g., ‘ant’ and ‘lion’). All conditions were randomly mixed within each block of trials.

### 2.7. Statistical analysis

*Distribution of visibility ratings*. We performed a Pearson chi-square test of independence to examine whether the distribution of visibility ratings differed between target- and prime-categorization trials (excluding catch trials). We then further examined the adjusted residuals within the crosstabulation (2 × 4 contingency table) indicating within-crosstab associations (Agresti, 2007). By examining adjusted residuals, we could identify which visibility ratings were more frequent on prime-categorization trials compared to target-categorization trials, and vice versa. Under the normal-distribution assumption, adjusted residuals larger than the 5% standard normal deviate of +1.96 (or smaller than –1.96) would indicate that the observed frequency count in a particular cell was significantly larger (or smaller) than would be expected if the null hypothesis were true, that is, if the distribution of visibility ratings on target- and prime-categorization trials did not differ (Agresti, 2007; Everitt, 1992).

*Target-categorization trials*. For each participant, any target-categorization trials with an RT deviating from the mean RT of its cell by more than 2.5 standard deviations was excluded as an outlier in all RT analyses. Reaction times were analyzed using linear mixed-effects models (LMM) analyses and accuracy data were analyzed using generalized linear mixed-effects model analyses (GLMM), an extension of LMM that allows categorical data analysis (Jaeger, 2008). All analyses were carried out using “R” statistical software (R Team, 2019). The model included response congruence (congruent vs. incongruent), visibility of the prime (0, 1, 2 and 3) as predictors (with an interaction term) and random subject-specific slopes and intercepts. This model is formally described as:


\[
RT\ \sim\ Congruence * Visibility + (Congruence * Visibility | Subject)
\]

Response priming refers to poorer performance on incongruent trials relative to congruent trials. For accuracy analyses, a GLMM for binary data was fitted by using the glmer function and a logit link function (Jaeger, 2008) with the same predictors as for the RT analyses. The summary output of the anova function for LMM models provides F-values. By contrast, the summary output of the GLMM function of the lme4 package provides p-values based on asymptotic Wald tests, which is common practice for generalized linear models (Bolker et al., 2009). We thus report F-values for response times and χ^2^-values for error rates.

*Prime-categorization trials*. Similar to the analysis of accuracy data on target-categorization trials, we analyzed accuracy using GLMM. The model included visibility as predictor and random subject-specific slopes and intercepts. This model is formally described as:


\[
Accuracy \sim Visibility + (Visibility|Subject)
\]

The intercepts represented the accuracy on 0-rated trials, and a significant intercept therefore indicated above-chance performance. We further report p-values for post-hoc comparisons following Tukey adjustments for multiple comparisons.

All effects were tested in a type *III ANOVA*, using lmer function of the lme4 package (Bates et al., 2015).

*Retrospective power analysis* To evaluate the power of our sample size, we performed a post-hoc power analysis for the effects of interest with an α of 0.05, using the simr package (Green & Macleod, 2016), and the powerCurve function in R to simulate the likelihood of finding these effects with different sample sizes.

## 3. Results

The data from eight participants (20%) met one criterion for exclusion: their percentages of 2- and 3- visibility ratings on catch (prime-absent) trials exceeded the group’s mean by > 2.5 standard deviations (i.e., >22.40%, M = 7.01%, SD = 6.16%). These participants were replaced with eight new participants. The distribution of the prime-visibility ratings is described in Table 1. Catch trials were excluded from all analyses and so were trials in which the prime and target were identical (i.e., “repetition trials”, 23.2% of all trials) – in order to avoid contamination of stimulus–response retrieval effects by simple perceptual priming.

**Table 1:** Distribution of the prime-visibility ratings for each trial type.


	0-VISIBILITY	1-VISIBILITY	2-VISIBILITY	3-VISIBILITY

Target-Categorization trials	46%	14%	12%	28%

Prime-Categorization trials	34%	16%	13%	37%

Catch trials	85%	8%	3%	4%


The Pearson Chi-squared analysis revealed that the distribution of visibility trials differed between target-categorization trials and prime-categorization trials, χ^2^(3) = 358.04, p < .001. Further analysis of adjusted standardized residuals revealed that the frequency of 0-visibility trials was higher on target-categorization trials, with an adjusted residual of 17.65, whereas the frequency of 1- and 3-visibility trials were higher on prime-categorization trials, with adjusted residuals of 3.29 and 15.57, respectively. The frequencies of 2- visibility ratings did not differ between the two trial types, with an adjusted residual of 1.24.

### 3.1. Target-Categorization Trials

Outlier-RT trials (1.87%) as well as error trials (11.11%) were excluded from all RT analyses. Visual inspection of residual plots did not reveal any obvious deviations from homoscedasticity or normality. Figure 2 shows response-priming effects for each visibility rating, separately for the 50% fastest trials and 50% slowest trials in each cell. Mean response times and error rates are presented in Table 2.

**Table 2:** Estimated marginal mean RTs (in milliseconds) and mean accuracy (in percentage) on target-categorization trials, as a function of response congruence (congruent and incongruent), prime visibility (0, 1, 2, and 3) and response speed (50% fastest and 50% slowest). Standard errors are presented in parentheses.


	FAST RTs (ms)	SLOW RTs (ms)	ACCURACY (%)

**3-visibility**			

Incongruent	571 (7.32)	751 (12.0)	86.9 (1.25)

Congruent	543 (6.93)	721 (11.6)	93.2 (0.92)

*Response priming*	28***	27***	6.3***

**2-visibility**			

Incongruent	564 (8.14)	720 (12.3)	92.9 (1.07)

Congruent	546 (8.36)	697 (13.1)	96.2 (0.88)

*Response priming*	18**	23*	3.3**

**1-visibility**			

Incongruent	544 (5.55)	698 (10.5)	93.8 (0.99)

Congruent	547 (7.21)	698 (12.1)	94.3 (1.18)

*Response priming*	*–3*	*0*	*0.5*

**0-visibility**			

Incongruent	524 (6.55)	667 (10.1)	89.3 (1.05)

Congruent	514 (8.34)	669 (10.6)	89.6 (0.89)

*Response priming*	10**	*–2*	*0.3*


*= p < .05, **= p < .01, ***= p < .001.

On fast trials, the main effect of congruence was significant, with slower RTs on incongruent than on congruent trials, F(1, 26.47) = 21.73, p < .001, and this effect interacted with visibility, F(3, 29.99) = 7.96, p < .001. Paired contrasts revealed that response priming was significant on 0-visiblity trials, t(36.2) = 2.93, p = .006, 2-visibility trials, t(30.6) = 2.85, p = .008, and 3-visibility trials, t(34.9) = 5.69, p < .001, but not on 1-visibility trials, t < 1.

On slow trials, the main effect of congruence was also significant, F(1, 25.92) = 9.68, p = .004, and interacted with visibility, F(3, 21.04) = 8.96, p < .001, indicating that primes elicited significant response priming on 2-visibility trials, t(28.7) = 2.65, p = .013, and on 3-visibility trials, t(31.6) = 4.61, p < .001, but not on 0- and 1-visibility trials, both ts < 1.

The accuracy results indicated no speed-accuracy trade-off. The main effect of congruence was significant, χ^2^(1) = 22.96, p < .001, and interacted with visibility, χ^2^(3) = 18.89, p < .001, indicating that response priming was significant on 2-visibility trials, χ^2^(1) = 7.18, p = .007, and 3-visibility trials, χ^2^(1) = 33.38, p < .001, but not on 0-visibility and 1-visibility trials, both χ^2^ < 1.

### 3.2. Prime-Categorization Trials

**Figure 3:**
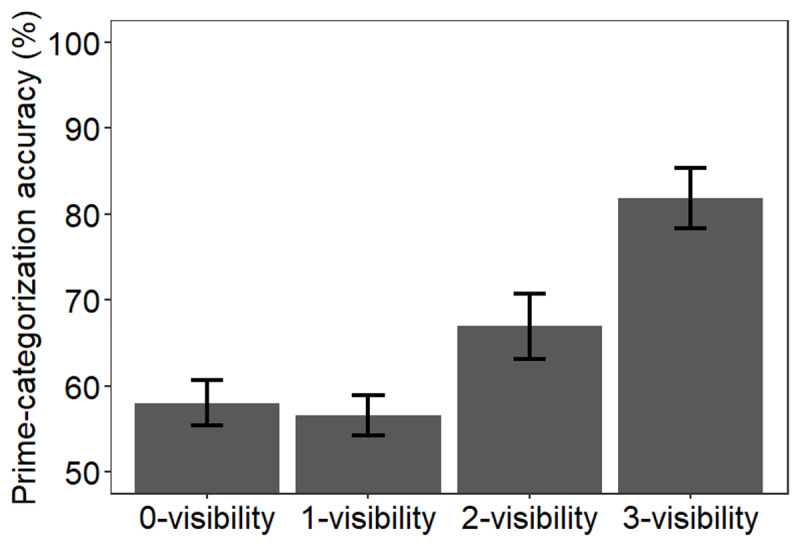
Mean accuracy (in percentage) on prime-categorization trials as a function of prime-visibility ratings. Error bars indicate standard errors.

The effect of visibility rating on prime-categorization accuracy was significant, χ^2^(3) = 29.78, p < .001 indicating that performance generally increased as the visibility rating increased, (see Figure 3), with an accuracy 58.0%, 56.6%, 66.9% and 81.9% for 0-, 1-, 2-, and 3-visibility trials, respectively. Follow-up analyses indicated that accuracy was similar for 0- and 1-visibility trials, χ^2^ < 1, and was higher on 2- than on 1-visibility trials, χ^2^(1) = 9.04, p = .01, and on 3- than on 2-visibility trials, χ^2^(1) = 18.88, p < .001. Accuracy was above chance for all four visibility ratings, all ps < .005, and crucially, on 0-visibility trials, χ^2^(1) = 9.27, p = .002.

### 3.3. Retrospective power analysis

We retrospectively estimated the power to detect response priming on RTs for each visibility rating. For fast-response trials, the power for 0-, 1-, 2- and 3-visibility trials was 81.5% (95% CI: 75.4%, 86.6%), 10.5% (95% CI: 6.6%%, 15.6%), 57.5% (95% CI: 50.3%, 64.4%), and 100% (95% CI: 98.2%, 100%), respectively. Further analyses revealed that to achieve 80% power, it would take 650 participants for 1-visibility trials and 60 for 2-visibility trials. For slow-response trials, the power for 0-, 1-, 2- and 3-visibility trials was 6.5% (95% CI: 3.5%, 10.9%), 10.5% (95% CI: 6.6%, 15.6%), 74.0% (95% CI: 67.3%, 79.9%), and 99.0% (95% CI: 96.4%, 99.9%), respectively. Further analyses revealed that to achieve 80% power, it would take 8000, 500 and 42 participants for 0-, 1- and 2-visibility trials, respectively.

We also estimated the power to detect above-chance prime-discrimination accuracy for each visibility rating. For 0-, 1-, 2-, and 3- visibility ratings, the power was 92.0% (95% CI: 87.3%, 95.4%), 84.0% (95% CI: 78.2%, 88.8%), 99.0% (95% CI: 78.2%, 88.8%), and 100% (95% CI: 98.2%, 100%), respectively.

## 4. Discussion

### 4.1. Summary of findings

Our results yielded three main findings. First, we successfully replicated the results reported by Micher and Lamy (2023, Exp. 1): we found response priming on both slow- and fast-response trials when participants reported consciously perceiving the prime (2- or 3-visibility ratings), but only on fast-response trials when participants reported no experience of the prime (0-visibility). This dissociation is inconsistent with the claim that participants were aware of the primes on 0-rated trials but miscategorized their subjective experience. Second, we found prime-discrimination performance to increase as visibility ratings increased, confirming that discrimination performance was correlated with conscious perception. Third and crucially, discrimination of 0-rated primes was better than expected by chance. Since the possibility that 0-rated trials were contaminated by conscious perception is unlikely in light of the dissociation between the response priming effects on fast and slow trials, this finding indicates that the blindsight-like performance observed here reflected unconscious processing. Therefore, our results suggest that relying on objective operational definitions of conscious perception (e.g., forced-choice discrimination performance) is misguided because performance on objective measures of conscious perception can be contaminated by unconscious processing.

### 4.2. Differences between prime- and target-discrimination trials

In the introduction, we argued that the prime should be processed in the same way in prime- and target-categorization trials because participants knew which task they should perform only after the prime had already disappeared. However, two findings suggest that the prime was processed differently in the two tasks: (1) prime-visibility ratings were higher than on prime- vs. target categorization trials and (2) the findings relative to 1-visibility ratings appeared to be inconsistent on prime- and on target-categorization trials. These findings are discussed next.

#### 4.2.1. Higher prime visibility on prime- vs. target categorization trials

Participants selected the 0-visibility rating less often and the 3-visibility rating more often during prime categorization relative to target categorization. Does this difference undermine our conclusions? In particular, is it possible that even though 0-visibility ratings were not contaminated by conscious perception on target-categorization trials, they were so on prime-categorization trials? The following arguments argue against this possibility.

There were two differences between the two tasks. The first is what appeared in the target display: an animal name, similar to the prime, or a “WWWWW” string. Participants should have been far less likely to misattribute conscious perception of the prime to the target on prime- than on target-categorization trials because a very dissimilar letter string replaced the target in the former. Thus, if anything, conscious perception should contaminate 0-visibility reports less often on prime- than on target-categorization trials (for which the dissociation between conscious and unconscious processing was demonstrated).

The second difference is whether participants were asked to categorize the target or the prime. Recent research has shown that cueing attention after a degraded stimulus is gone can retrospectively trigger conscious perception of that stimulus (e.g., Sergent et al., 2013). For instance, Sergent et al. (2013) showed that high-visibility ratings were more frequent and low-visibility ratings were less frequent when a retro-cue appeared at the same location as the critical stimulus than when it appeared at the alternative location. Based on these findings we speculate that here, the response cue (“WWWWW”) triggered retrospective allocation of attention to the prime (because it instructed participants to discriminate this prime). As a result, participants were more likely to become aware of primes on prime-categorization trials than on target-categorization trials.^5^ In this case, there is no reason to assume that participants used 0-visibility ratings differently in the two tasks.

Note that if participants were more aware of the prime on prime-categorization trials (compared to target-categorization trials) due to retrospective influence of attention, it would extend previous findings in two important ways. First, they would indicate that attention can retrospectively rescue visual stimuli from invisibility not only for low-contrast stimuli but also for masked stimuli. Second, they would indicate that retrospective attention increases access to conscious perception not only when allocated in space but also when allocated in time. Indeed, in our study unlike in Sergent et al.’s (2013) all stimuli were presented at the same, central location, and we surmise that the stimulus that appeared last (WWWWW or a target) determined whether attention would be retrospectively directed to the prime event or to the more recent target event.

#### 4.2.2. The status of 1-visibility trials

The second difference between prime- and on target-categorization trials concerns 1-visibility trials. For that rating, there was no response priming on target-categorization trials,^6^ but discrimination performance was nevertheless better than chance on prime-categorization trials.

Informal post-experimental reports collected in our lab suggest that participants tend to use the 1-visibility rating when they are “off-task” (e.g., when they blinked or were inattentive). On these “lapse trials”, then, participants do not process the primes, and therefore response priming on 1-visibility trials is underestimated. By this account, prime-discrimination performance was also underestimated in our study. Because we speculate that 1-visibility trials reflect a mixture of off-task trials, in which performance was presumably at chance, and on-task trials in which the prime was processed, this account would explain why prime-discrimination performance tended to be lower than on 0-visibility trials instead of higher, as typically found in previous studies investigating blindsight-like performance (e.g., Ramsøy & Overgaard, 2004; Szczepanowski et al., 2013; Wierzchoń, Paulewicz, et al., 2014; but see Overgaard et al., 2008). However, it is not clear why 1-visibility ratings might be less contaminated by attentional lapses in those studies than in studies relying on the liminal-prime paradigm (e.g., Avneon & Lamy, 2018; 2019; Kimchi et al., 2018; Micher & Lamy, 2023; Ophir et al., 2018). Further research is required to clarify how participants use 1-ratings in different experimental conditions.

### 4.3. The criterion problem using PAS

Overgaard et al. (2006: 2008) showed that when observers rate a stimulus as unseen, their objective performance at categorizing a property of that stimulus is higher when their subjective experience is indexed via a binary seen/not-seen measure than via a multi-level report scale such as PAS. Thus, by allowing participants to report faint experiences with appropriate labels such as “brief glimpse” or “almost clear image”, they demonstrated that PAS minimizes contamination of “unawareness” reports by conscious perception.

Nevertheless, some argue that PAS still suffers from the criterion problem. For instance, Fahrenfort et al. (2024) demonstrated that various manipulations, such as changing the wording of instructions or imposing different penalties on ‘miss’ and ‘false-alarm’ trials, affect the distribution of subjective reports and thus the extent of measured unconscious-processing effects. They concluded that criterion shifts confound measures of unconscious processing under post-hoc trial sorting. It is of course unsurprising that manipulating non-perceptual factors impacts the ratings participants use, even when explicitly instructed to respond solely based on their experience, as in Fahrenfort et al.’s (2024) study: participants weigh which information should guide their behavior, and the incentive to avoid punishment likely outweighs the desire to follow instructions exactly. In other words, it is impossible to determine whether in the absence of additional instructions, participants may favor one PAS rating over the others.

Here, we claimed that the criterion cannot account for the significant unconscious response priming observed in our study, because it was dissociated from conscious response priming; it occurred only on fast trials, whereas conscious response priming occurred on both fast and slow trials. However, an alternative account is that participants may be more likely to report being unaware of the prime when they respond fast than when they respond slowly. Consequently, they may be more likely to mislabel primes as “unaware” on fast- than on slow-response trials, even though they are in fact aware of these primes.

Two arguments make this possibility unlikely. First, this alternative account predicts that on catch trials, responses to the target should be faster on 0-visibility trials than on 3-visibility trials – which did not happen here. It also predicts that when participants are not asked to provide a subjective report after the target-categorization task, the dissociation between fast and slow trials should be absent. Yet, the same pattern of results was observed in a seminal subliminal-priming experiment where the prime was assumed to be objectively “unaware” (see Figure 2b in Dehaene et al., 1998).

An intriguing avenue for further research would be to examine how shifting the criterion would affect the dissociation between unconscious and conscious response priming over the RT distribution. We predict that if participants are encouraged to adopt a conservative criterion and therefore select the 0-visibility rating even on trials in which they have some conscious percept of the prime, the dissociation should weaken: response priming on 0-visibility trials should also become apparent on slow trials.

We further suggest that PAS is less susceptible to the criterion problem in the liminal-prime paradigm than in other paradigms. In particular, with stimuli that are so degraded that the lowest subjective rating is used on an overwhelming majority of trials (e.g., Mudrik & Koch, 2013; Soto et al., 2011), participants may habitually respond that they did not see the stimulus and select the lowest visibility rating even when they have some conscious percept the stimulus. By contrast, with liminal primes, participants use the ratings in similar proportions and therefore do not develop a habitual response.

## 5. Conclusions

By showing that unconscious processing can co-occur with above-chance performance our findings have three main implications. First, they indicate that objective forced-choice discrimination performance is contaminated by unconscious processing. As such, they complement earlier criticisms raised against the standard subliminal-priming procedure (e.g., Lin & Murray, 2014; Pratte & Rouder, 2009): they suggest that using chance discrimination performance as the only valid indicator of unawareness can underestimate unconscious processing. Second, they show that subjective reports on a multi-level scale such as PAS can provide a valid measure of conscious perception. Specifically, we show that demonstrations of unconscious processing using these measures cannot be entirely accounted for by criterion shifts. Finally, our findings showcase how useful the liminal-prime paradigm can be to uncover dissociations between conscious and unconscious processes.

## Data Accessibility Statement

All data and analysis scripts are available on figshare: doi.org/10.6084.

## Footnotes

We use the term “blindsight-like performance” to refer to above-chance discrimination of subjectively invisible stimuli among healthy participants, that is, participants without any neurological lesion (Hesselmann et al., 2011).

In other words, the comparison between seen and unseen primes is conducted on physically identical stimuli, rather than between masked and unmasked stimuli as in many previous studies (e.g., Cheesman & Merikle, 1986; Faivre & Koch, 2014; Kiefer & Spitzer, 2000).

The followed the OSF pre-registration in all its aspects except for one: instead on restricting our analysis to 0- and 3-visibility trials, we included all visibility ratings in the analysis. The reason for this change is that the model with all four levels typically does not converge but it did converge in the present experiment. We therefore opted for a more complete report of the data. The results and conclusions remain the same when intermediate levels are removed.

This scale differs from Ramsoy and Overgaard’s (2004) and is identical to the scale used by Micher and Lamy (2023) with the same stimuli. As recognized by Sandberg & Overgaard (2015), it is important to tailor the specific descriptions of PAS rating categories to the stimuli and discrimination of interest.

Interestingly, this putative retro-cue effect followed the same pattern as in Sergent et al.’s (2013) study (see also Sergent, 2018; Thibault et al., 2016): the proportions of invisible and clearly seen primes were strongly affected, whereas intermediate ratings were much less affected, if at all.

It is noteworthy that the finding that response priming is absent on 1-visibility trials but present on 0-, 2-, and 3-visibility trials has been reported before (e.g., Kimchi et al., 2018; Ophir et al., 2018), but only speculations were put forward to account for it. For instance, Kimchi et al. (2018) suggested that inhibition of the primed response for barely visible stimuli thought to underlie negative response priming (e.g., Eimer & Schlaghecken, 2003) might account for the null response priming found on 1-visibility trials.
